# Dual graft living donor liver transplantation – a case report

**DOI:** 10.1186/s12893-019-0606-5

**Published:** 2019-10-22

**Authors:** Nikam Vinayak, Mohanka Ravi, Golhar Ankush, Bhade Rashmi, Rao Prashantha, Gadre Parul, Shrimal Anurag

**Affiliations:** Institute of Liver Diseases, HPB Surgery and Transplantation, Global Hospital, 35, Dr. E Borges Road Opp. Shirodkar High School, Parel, Room No- 202, 2nd Floor, Mumbai, Maharashtra 400012 India

**Keywords:** Living donor liver transplantation, Deceased donor liver transplantation, End stage liver disease, Graft to recipient weight, Small-for-size syndrome, Future liver remnant

## Abstract

**Background:**

Living donor liver transplantation (LDLT) has emerged as an equally viable option to deceased donor liver transplant for treating end stage liver disease patients. Optimising the recipient outcome without compromising donor safety is the primary goal of LDLT. Achieving the adequate graft to recipient weight ratio (GRWR) is important to prevent small for size syndrome which is an uncommon but potentially lethal complication of LDLT.

**Case presentation:**

Here we describe a case of successful dual lobe liver transplant for a 32 years old patient with ethanol related end stage liver disease. A right lobe graft without middle hepatic vein and another left lateral sector graft were transplanted successfully. Recipient and both donors recovered uneventfully.

**Conclusion:**

Dual lobe liver transplant is a feasible strategy to achieve adequate GRWR without compromising donor safety.

## Background

Liver transplantation is a gold standard treatment for a patient with end-stage liver disease.

Living donor liver transplantation (LDLT) rate is increasing due to the shortage of deceased liver donations. Small for size syndrome and small donor liver remnant are two uncommon but potentially lethal complications of LDLT [1].

Small for size syndrome (SFSS) generally occurs when the graft recipient weight ratio (GRWR) is less than 0.8. There is disequilibrium between increased metabolic demand and liver regeneration which leads to a severe graft dysfunction [2]. The clinical presentation of SFSS is intractable ascites, coagulopathy, and jaundice [2, 3]. The most accepted pathophysiological mechanism for SFSS is over-perfusion of transplanted liver graft [3].

LDLT is the most common type of liver transplant in India. Almost 33% of potential live donors are rejected as liver donors for adult recipients because of calculations suggesting a small for size (SFS) graft, a small future liver remnant (FLR) and steatosis [4].

In recent years the subset of morbidly obese patients undergoing liver transplant is raising due to the increase in the incidence of non-alcoholic steatohepatitis (NASH) related end stage liver disease (ESLD). In order to overcome the problems of SFSS, optimise donor safety and increase the donor pool, dual graft liver transplant is the best feasible option. The first successful dual lobe LDLT using left lobe graft and left lateral section was performed by Lee et al. in year 2000 [5]. In this paper we are presenting a case of ethanol induced ESLD patient who successfully underwent dual graft LDLT by using right lobe and left lateral segment (LLS) grafts.

## Case presentation

### Recipient details

The recipient was a 32 years old male (weight-90 kg, height − 165 cm, BMI-32 kg/m^2^) diagnosed with ethanol related ESLD, decompensated with jaundice, ascites, and hydrothorax (MELD Score- 34, CTP Score − 11/C) (Table 1). He was evaluated as per our standard recipient evaluation protocol and placed on the deceased donor waiting list. Liver anatomy was evaluated by computed tomography (CT) triple phase abdomen which showed features of chronic liver disease with portal hypertension with a patent portal vein without any space occupying lesion in the liver. The patient was counselled for LDLT in view of a long waiting list for deceased donor liver transplant (DDLT). The patient needed dual lobe living donor liver graft due to inadequate partial liver graft volumes of individual donors.

**Table 1:** Clinical Data of Recipient

Age	32 years
Gender	Male
Blood group	O positive
Weight	90 kg
Height	165 cm
BMI	32 kg/m^2^
CTP Score	11/C
MELD	34
Alfa fetoprotein (AFP)	4.3 ng/ml

### Donor details

Donors were found to be fit for donor hepatectomy after clinical examination and laboratory tests (Table 2).

**Table 2:** Donor Details

Details	Donor I	Donor II
Graft Type	Rt Lobe without MHV	Lt Lateral Graft
Relation	Sister	Uncle
Age	29	49
Gender	Female	Male
Blood Group	O positive	O positive
BMI (kg/m^2^)	22.9	23.4
CT LAI	+ 11	+ 7.8
MR Elastography (mean shear stiffness and Fat Fraction)	2.2 kPa, 2–4%	2.4 kPa,2–4%
MRCP- Bile Duct classification	Huang A_4_B_1_	Huang A_1_B_1_

### Donor liver Volumetry and anatomical details

#### Donor I

**Fig. 1:**
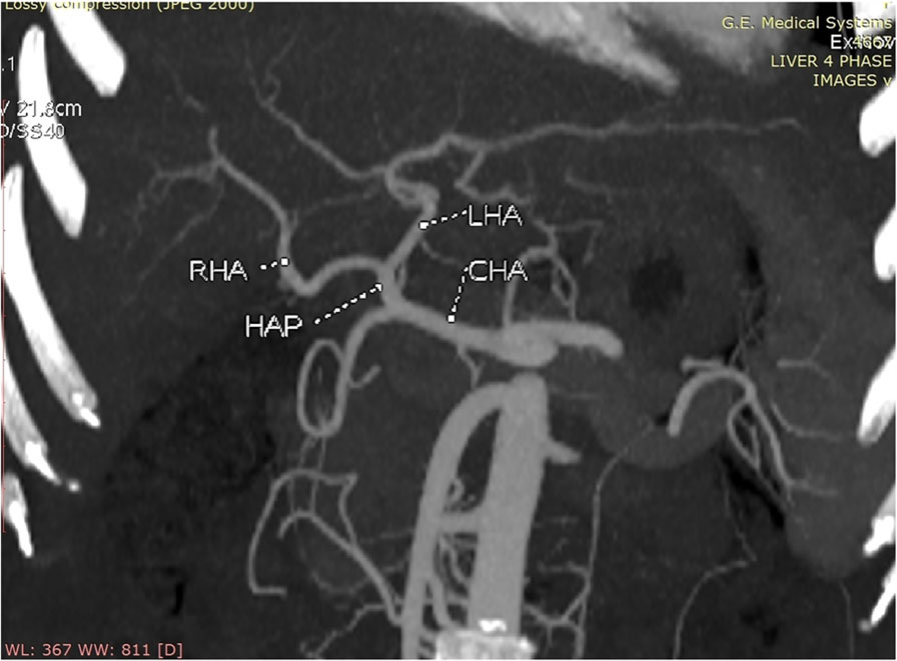
Donor I - Hepatic Arterial Anatomy

**Fig. 2:**
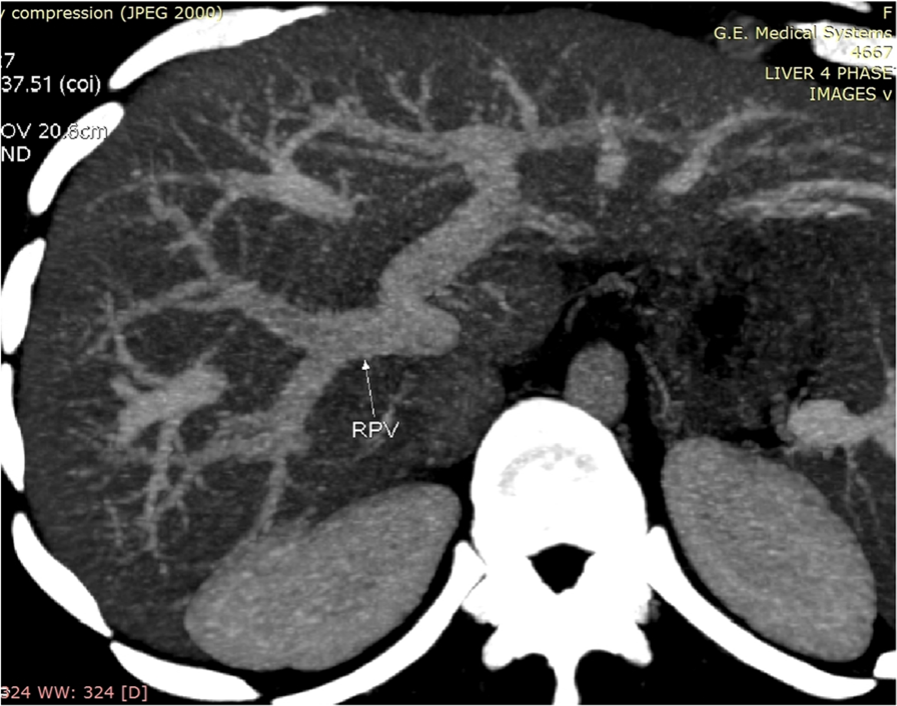
Donor I - Portal Vein Anatomy

**Fig. 3:**
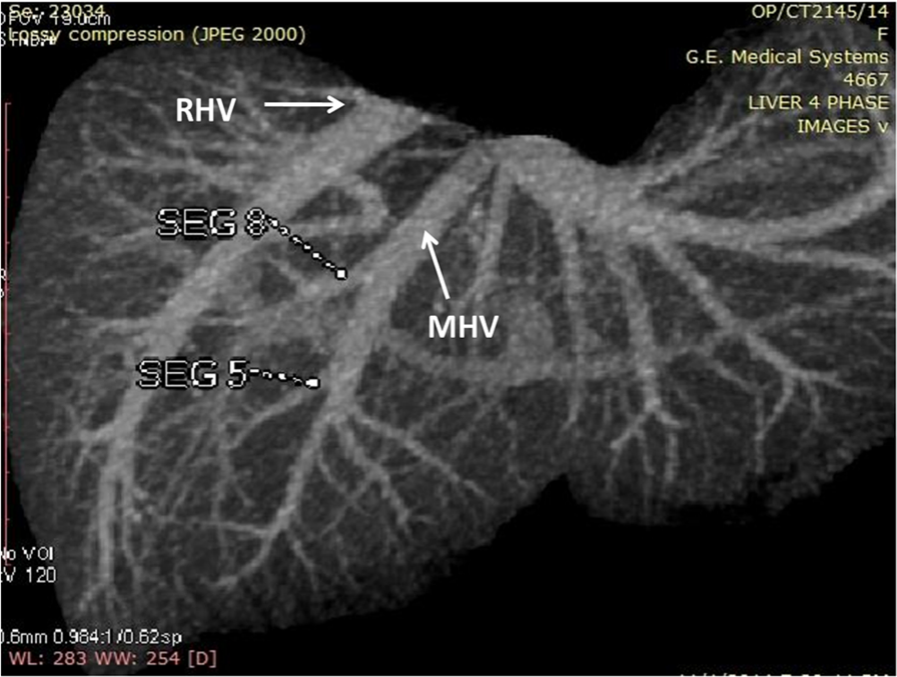
Donor I - Hepatic Venous Anatomy

**Fig. 4:**
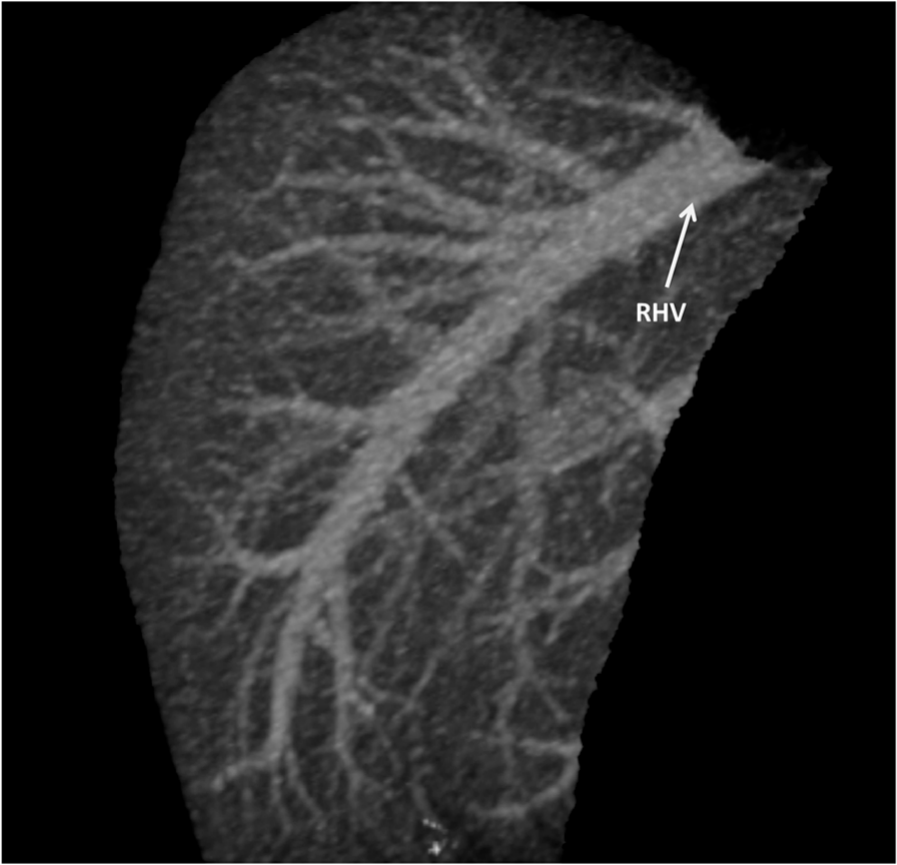
Donor I Planned Right Lobe Without MHV Graft

**Fig. 5:**
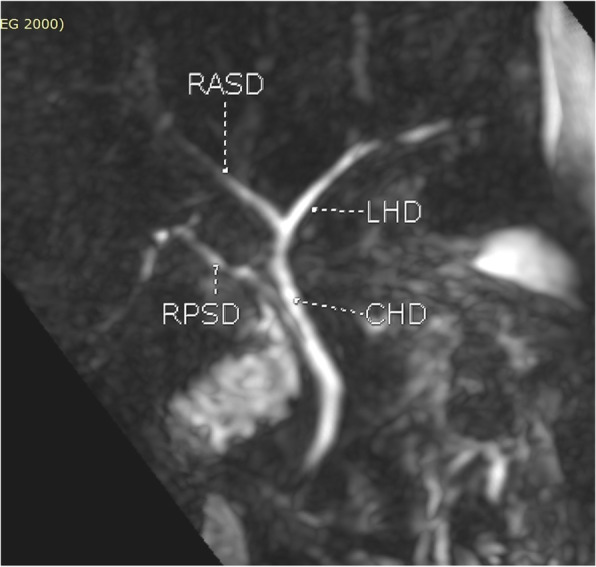
Donor I- Bile Duct Anatomy (Huang Type A4B1)

- **Hepatic Artery**: Hepatic artery proper was dividing into the left hepatic artery (LHA) and right hepatic artery (RHA). RHA had an extra-hepatic length of 27 mm. Segment 4 artery was arising from LHA and there were no other accessory arteries identified (Fig. 1).
- **Portal Vein**: Portal vein anatomy was Nakamura Type A (Fig. 2). The length of right portal vein was 11 mm. The significant crossover of the portal drainage was absent.
- **Hepatic Veins**: Two inferior right hepatic veins measuring 4.1 mm and 4.5 mm in diameter were noted draining into inferior vena cava. Significant size segment V (3.4 mm) and VIII (2.8 mm) veins were draining into MHV (Figs. 3 and 4).
- **Bile Duct** – Bile duct anatomy was Huang Type A_4_B_1_ (Fig. 5).

#### Donor II

**Fig. 6:**
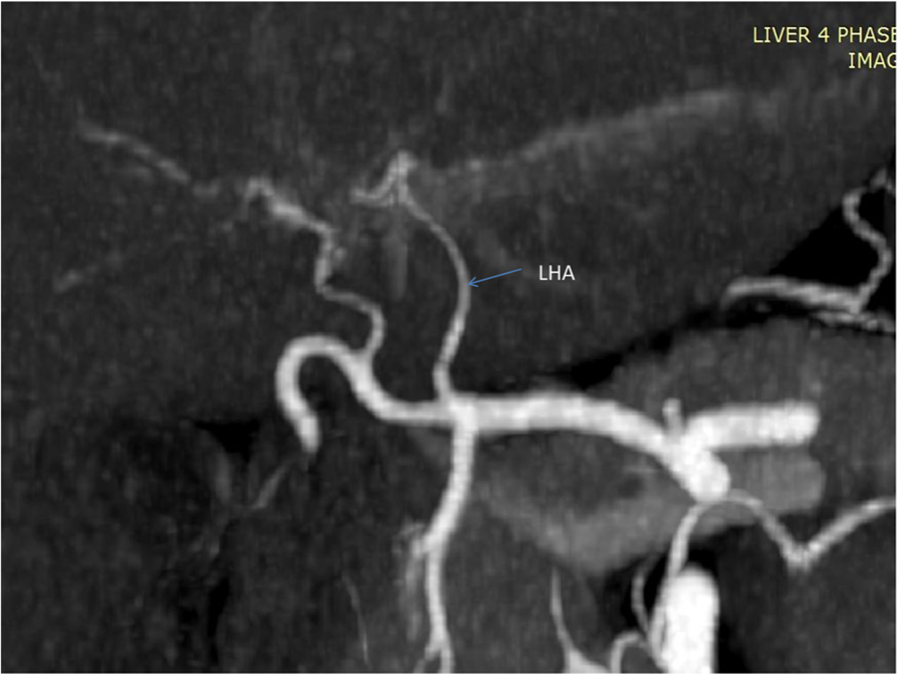
Donor II - Hepatic Artery Anatomy

**Fig. 7:**
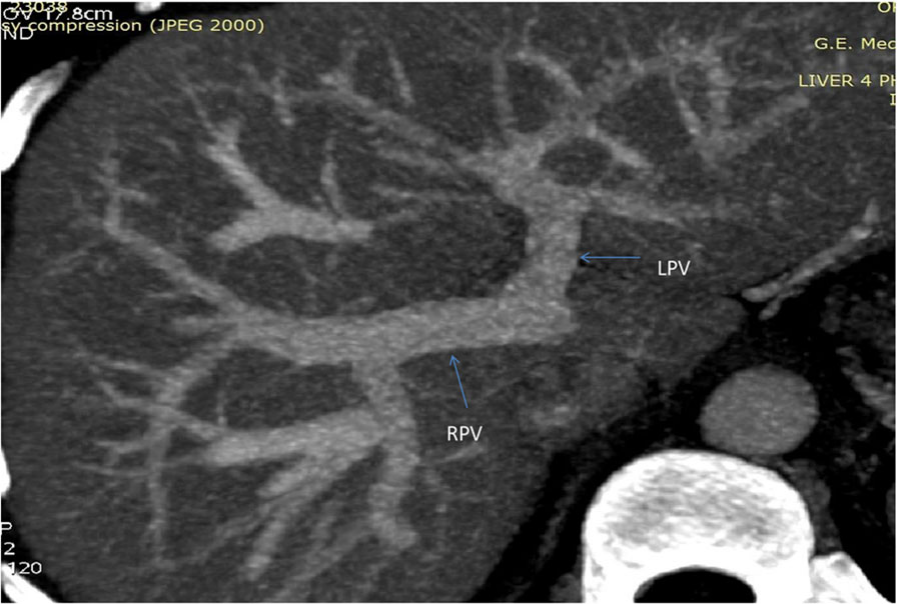
Donor II - Portal Vein Anatomy

**Fig. 8:**
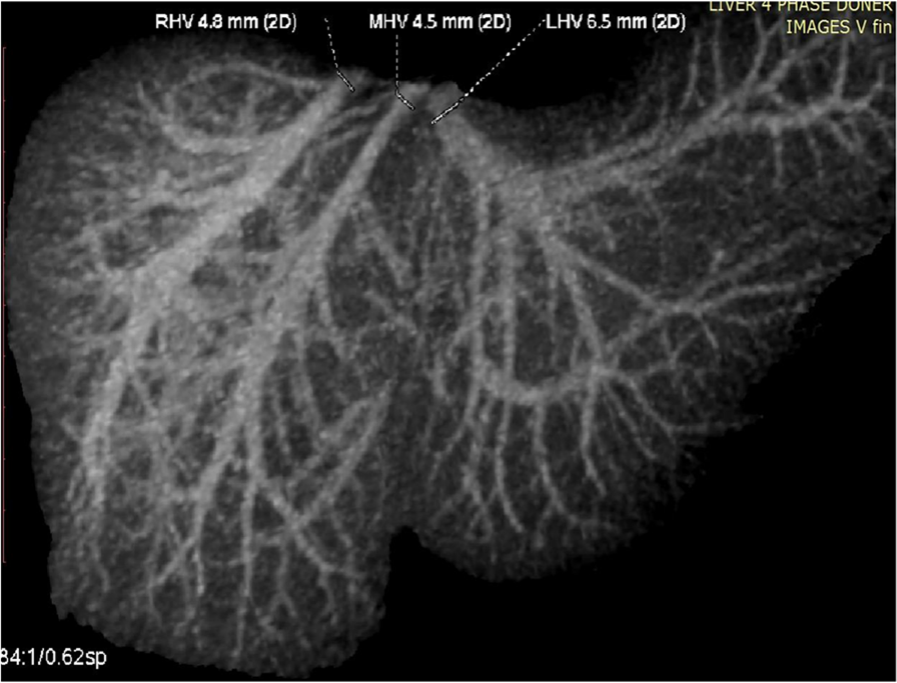
Donor II - Hepatic Venous Anatomy

**Fig. 9:**
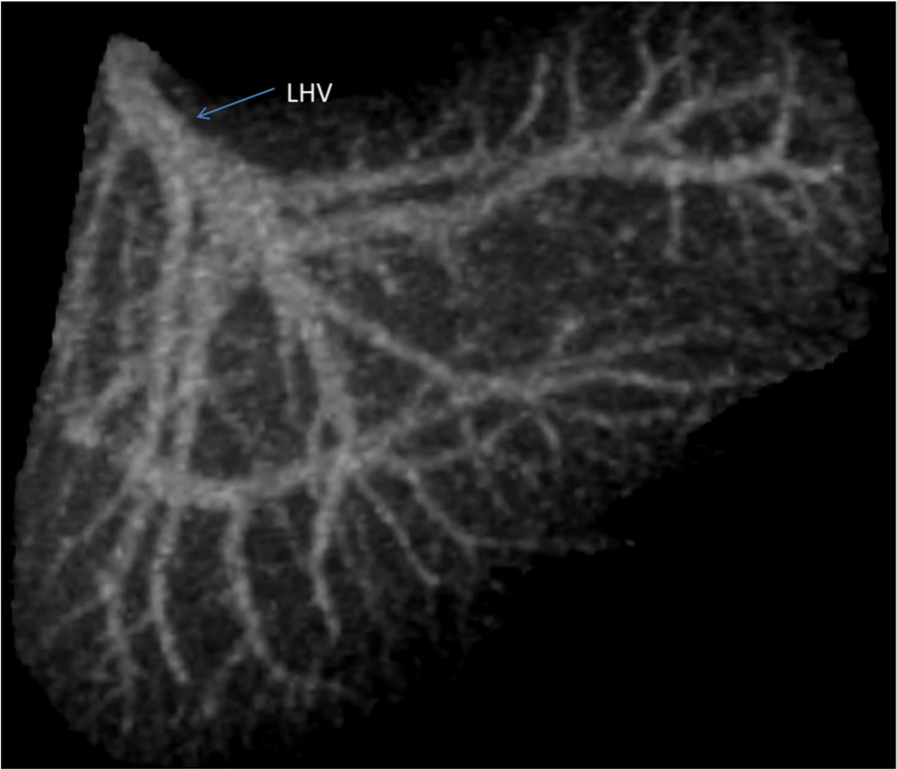
Donor II Planned Left Lateral Sector Graft

**Fig. 10:**
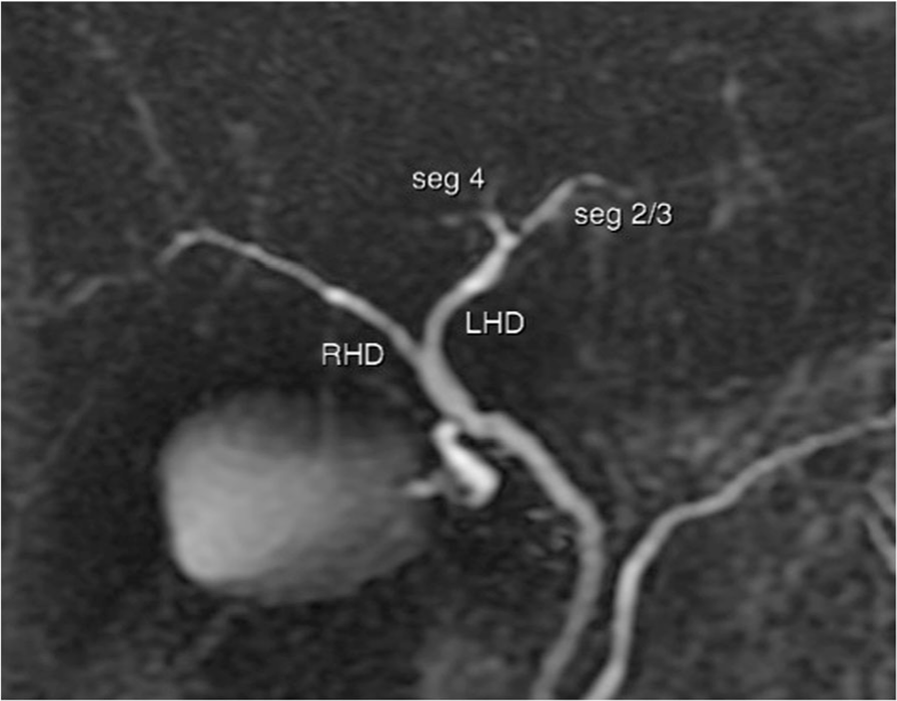
Donor II Bile_Duct Anatomy (Huang Type A1B1)

- **Hepatic artery**: Hepatic artery proper (HAP) was dividing into LHA and RHA. LHA was arising from HAP immediately at its origin and supplied segments 2 and 3. Segment 4 artery was arising from RHA (Fig. 6).
- **Portal Vein**: Portal vein anatomy was Nakamura Type A (Fig. 7). Segment 4 portal vein (measuring 4.4 mm) was arising from the left portal vein. No significant crossover of the drainage was noted.
- **Hepatic veins**: There was a short common channel between left hepatic vein (LHV) and Middle hepatic vein (MHV) measuring 5.8 mm in length (Figs. 8 and 9). Left hepatic vein measured 6.5 mm near its ostium. A segment II/III vein measuring 9 mm in diameter was joined by segment III vein measuring 4.5 mm in diameter to form LHV. Another segment III vein (3 mm diameter) joined the LHV.
- **Bile Duct** – Bile duct anatomy was Huang Type A_1_B_1_ (Fig. 10).

CT Volumetric analysis of either donor livers showed a relatively small right liver lobes and calculated graft recipient weight ratios (GRWR) were 0.63 and 0.62 respectively. As neither of the two probable donors were suitable as a single donor (Table 3), the decision was made to perform LDLT using dual liver grafts. In this case, we chose the right liver lobe of Donor I together with the left lateral Sector of Donor II to get an adequate GRWR. Future liver remnants (FLR) were adequate in both donors (donor I − 39% and Donor II-70%).

**Table 3:** Donor CT volumetric graft planning

Graft Type	Donor I	DonorII	Feasibility
TLV	941 cc	986 cc	
Right Lobe with MHV	589 cc	592 cc	
Remnant	352 cc (37%)	394 cc (39.9%)	
GRWR	0.65	0.65	No
Right Lobe without MHV	567 cc	561 cc	
Remnant	374 cc (39%)	425 cc(43%)	
GRWR	0.63	0.62	No
Left lobe	374 cc	425 cc	
Remnant	567 cc(60%)	561 cc(57%)	
GRWR	0.42	0.47	No
Left Lateral Lobe		295 cc	
Remnant		70%	
GRWR	–	0.32	No
Dual Lobe = (Donor I) Right lobe without MHV + (DonorII) Left Lateral	567 cc	295 cc	
Lobe			
GRWR	0.63	0.32	YES (0.95)

### Surgical procedure

Risks and benefits of the surgery were discussed and informed consents were obtained from both donors and recipient. Recipient and both donor surgeries were started simultaneously.

### Recipient hepatectomy

Surgical access was through a right sub-costal incision extended vertically in the midline till the xiphoid process. Recipient’s cirrhotic liver was explanted as per standard hepatectomy procedure. Recipient received a 10 mg/kg dose of injection methylprednisolone immediately after liver explantation.

### Donor graft harvesting

Right lobe without MHV graft was harvested from Donor I after the division of right anterior and posterior sectoral ducts (RASD and RPSD), right hepatic artery (RHA), right portal vein (RPV), right hepatic vein (RHV), and right inferior hepatic vein (RIHV). Left lateral lobe graft was harvested from Donor II after the division of the left hepatic duct, left hepatic artery (LHA), left portal vein (LPV) and LHV.

**Fig. 11:**
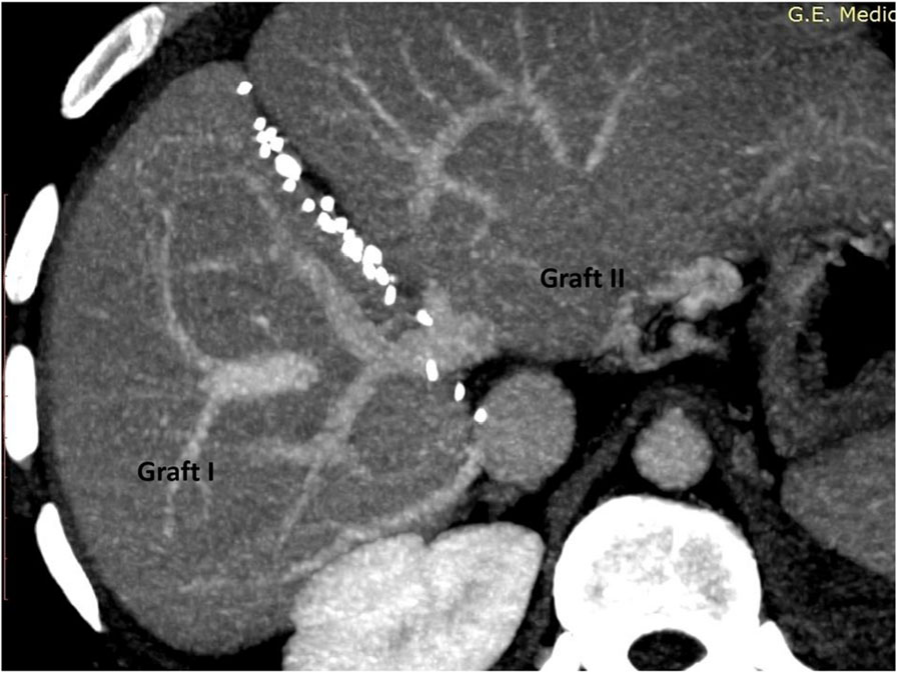
Dual Graft I (Right lobe and Left latera l sector)

Actual harvested graft weights were 503 g (right lobe without MHV graft) and 374 g (Left lateral sector graft) (Fig. 11) giving GRWRs of 0.55 and 0.41 respectively (Total GRWR 0.96) (Table 4).

**Table 4:** Dual lobe –Actual graft weight and GRWR

Donor Type	Donor I	Donor II	Dual Lobe
Graft Type	Right Lobe without MHV Remnant	Left Lateral Lobe	Right lobe without MHV + Left Lateral Lobe
Actual graft weight (Gm)	503 gm	374gm	877 gm
GRWR	0.55	0.41	0.96

Operative time for Donor I and Donor II hepatectomy was 360 min and 310 min respectively with a blood loss of 450 ml and 370 ml.

### Back table preparation

Grafts were perfused with UW solution. As hepatic vein tributaries of segment V and VIII were less than 3.5 mm diameter, venous drainage of these segments was not established. In both grafts, no other back table reconstruction was needed.

### Sequential manner implantation of dual grafts

#### Graft I- right lobe implantation and vascular anastomosis

- The right lobe without MHV graft was orthotopically implanted on the right side of IVC.
- IVC was clamped using a side biting clamp. Cavotomy was performed at the orifice of RHV.
- **Hepatic venous anastomosis**
  Graft RHV was anastomosed to recipient IVC in an end to side manner with 5–0 polypropylene continuous suture.
  Graft RIHV was anastomosed to recipient IVC in an end to side manner with 6–0 polypropylene continuous suture.
- **Portal Venous anastomosis**
  Graft RPV was anastomosed to recipient RPV (end to end manner) using a 6–0 polypropylene continuous suture with appropriate growth factor.
  Graft reperfusion was immediate and uniform.
- **Arterial anastomosis**:
  Graft RHA anastomosed with recipient RHA with 8–0 polypropylene interrupted suture under three time’s magnification.
  After arterial reperfusion intra-operative doppler ultrasound showed regular portal venous, hepatic venous and hepatic arterial flow.

#### Graft II- left lateral lobe implantation and vascular anastomosis

**Fig. 12:**
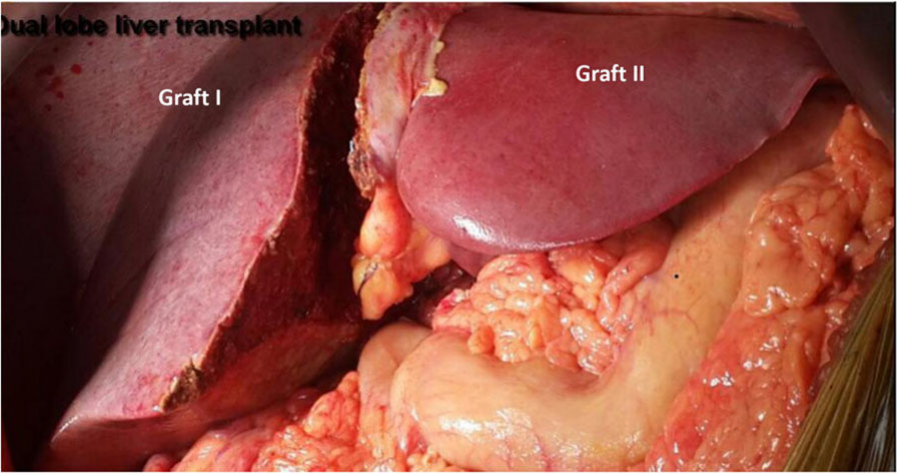
Implanted Dual Grafts

- **Hepatic venous anastomosis**
  Graft LHV was anastomosed to cavotomy made on recipient IVC at the orifice of LHV and MHV (end to side manner) with 5–0 polypropylene continuous suture.
- **Portal Venous anastomosis**
  Graft LPV was anastomosed to recipient LPV (end to end manner) using a 6–0 polypropylene continuous suture
  Graft reperfusion was immediate and uniform.
- **Arterial anastomosis:**
  Graft LHA anastomosed with recipient segment IV hepatic artery with 8–0 polypropylene interrupted suture under three times magnification.
  After arterial reperfusion intra-operative doppler ultrasound showed regular portal venous, hepatic venous and hepatic arterial flow in the left lateral graft as well.
- **Biliary anastomosis**
  Number of graft ducts: two in right lobe graft (RASD and RPSD) and 1 in left lateral graft
  Number of anastomosis: 3
  Anastomosis no 1: Graft RASD was anastomosed to the recipient right hepatic duct (RHD) with 6–0 Polydioxanone in an interrupted manner.
  Anastomosis 2: Graft RPSD to the recipient cystic duct anastomosed with 6–0 Polydioxanone in an interrupted manner.
  Anastomosis 3: Graft left hepatic duct (LHD) to the recipient LHD with 6–0 Polydioxanone in an interrupted manner. (Fig. 12).

### Post-operative course

#### Recipient

Recipient recovered well postoperatively. He received standard triple immunosuppressant. The patient was discharged on the eleventh postoperative day with normal liver graft function and good general condition.

#### Donor

Both donors recovered well postoperatively. They were discharged in good general condition and with normal liver function tests.

#### Follow up

The recipient was followed up weekly for the first 2 months and then every monthly. During follow up the patient was examined clinically and by laboratory tests including liver function tests, renal function tests, and serum tacrolimus level. A radiological assessment of hepatic vasculature was done whenever indicated. The long-term outcome, evaluated after a 48-month follow-up, was optimal with normal graft function, good general condition and superior quality of life.

## Discussion and conclusion

There is an increasing incidence of ESLD and shortage of deceased donor organ pool especially in East Asian countries including India. LDLT has emerged as a crucial solution to tackle the situation of disparity between demand and supply of liver allografts. The evolution of LDLT is a history of search for an optimal graft to minimize donor risk and maximize recipient outcome simultaneously [6].

It has been shown that minimum GRWR of 0.8 is required to reduce the occurrence of SFSS [7]. Also most transplant surgeons accept a cut off of atleast 30% remnant liver volume to optimize the donor’s own safety. Undoubtedly, while planning LDLT, the safety of donors must have great importance and unavoidable risk for the donor must be balanced against the potential benefit for the recipient. For this reason, appropriate donors should provide a sufficient GRWR for the recipient while having a secure FLR after donation [8]. To overcome these problems, especially in large size recipients, dual lobe LDLT can be a feasible and realistic option [9].

### Ethical dilemma and drawbacks of dual lobe

The surgical risk to the donor is always an ethical dilemma even with increasing application and success of LDLT. Donor death has an immense impact on the surviving recipient, other family members, and the transplant team. This ethical dilemma is compounded in dual lobe LDLT as two donors are at risk.

Left lobe donor hepatectomy is associated with lower morbidity and mortality than right lobe donor hepatectomy. Dual left lobe LDLT with two minor left lobe donor hepatectomies may actually have less combine donor risk than one major right lobe donor hepatectomy [10]. While donor mortality was estimated to be approximately 0.4 to 0.5% after right hepatectomy, risk of death for donors of a left lateral sector hepatectomy is 0.1% [11]. For this reason, the left lobe grafts (LL or LLS) are preferred over right lobe graft in dual lobe LDLT [12].

In the present case, it could have been possible to use reversed left lobe graft. However, we chose the Right lobe graft over the left lobe as we have maximum experience in Right Lobe LDLT. We have 100% safety record for all our Right lobe donors (220 Cases). Moreover, reverse left dual graft surgery is technically challenging and associated with more complications.

The issue of donor risk can be partially defeated by expert dual lobe transplantation teams, technical advances and deliberately choosing the dual left lobe grafts or accepting the low GRWR single lobe graft with inflow modulation techniques like splenectomy, splenic artery ligation or porto-caval shunt. In our institute we prefer to use a porto-caval shunt if intra-operative actual GRWR is less than 0.7 [13]. Higher cost of the procedure is another drawback of the dual lobe LDLT, but this actually may be offset by the higher morbidity associated with SFSS, leading to cost escalation in SFS graft LDLT recipients.

In this case, there is a disparity between volumetric estimated graft weight (EGW- 295 g) and actual graft weight (AGW- 374 g) of the left lateral segment of donor II. Right and left lobes EGW on CT Volumetry is overestimated as compare to AGW. For the Left lateral segment (LLS) graft, CT may underestimate the volume because the actual surgical plane of transection is around 1 cm to the right of a falciform ligament whereas radiological plane is at falciform ligament. In addition to that non-hepatic tissues like falciform ligament which are excluded in CT volumetry are a part of LLS allograft included in AGW [14].

### Difficulties in surgical planning and decision making

Surgical planning and decision-making process are complex and difficult in dual graft LDLT. Both donor and type of graft selection are very crucial for the optimal outcome. In our institute, we investigate donor as per protocol which includes laboratory investigations, estimation of liver attenuation index (LAI) on computed tomography, triphasic CT abdomen with the estimation of liver volumes. We asses biliary anatomy with MRCP preoperatively and with intra-operative cholangiogram.

**Fig. 13:**
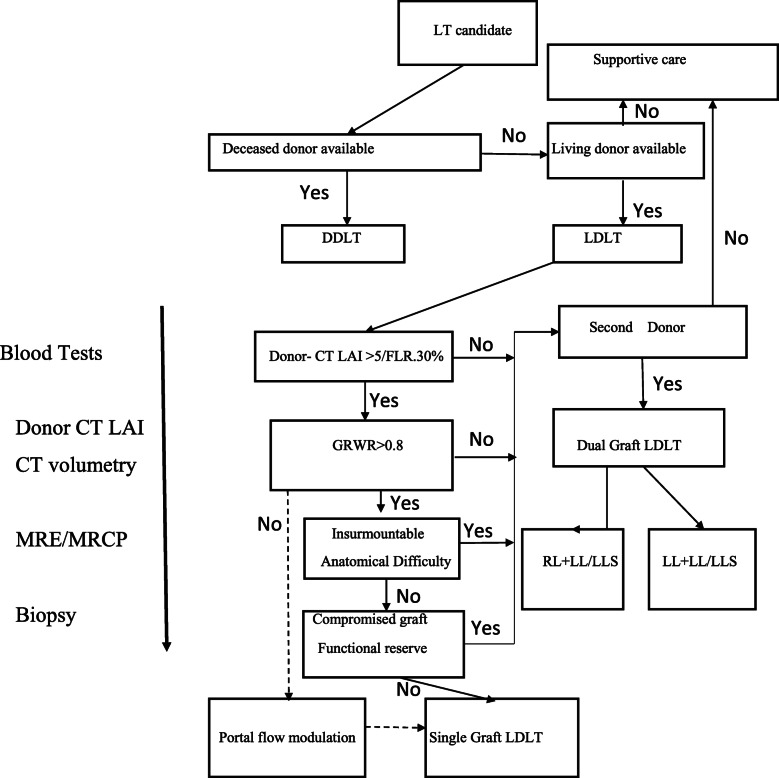
Dual Lobe Decision making Protocol

Decision-making process of dual graft LDLT needs to be standardized and to achieve consensus across the transplant community, Yinzhe Xu et al. have proposed a decision making pathway for same [15] (Fig. 13). The implantation of two grafts requires huge technical precision like complex hepatic venous outflow reconstructions, tailored use of a cryopreserved interposition vein graft and anastomosis of multiple hepatic arteries, which are challenging and pivotal element of dual graft LDLT. Before implantation of the liver grafts, venoplasty of the hepatic veins in the grafts and the recipient should be performed to make wide outflow orifices and prevent outflow narrowing [16].

Post dual graft LDLT biliary complications incidence is around 21%. Biliary reconstruction of both grafts can be performed using duct to duct anastomosis or combination of duct to duct and hepatico-jejunostomy (HJ) to each graft. A biliary reconstruction strategy is therefore critical to the outcome of dual graft LDLT as well. Dual duct-to-duct anatomoses are preferred over HJ for being more physiologic and allowing better access for post-transplant endoscopic and interventional radiological procedures [17].

Furthermore, unnecessary dissection of recipient bile ducts in the hilar plate should be avoided and as much as connective tissue should be preserved to avoid ischemic biliary damage. Dual graft LDLT is an extremely complex procedure, its outcome relies on the appropriate patient and donor selection, detailed donor evaluation, precise surgical planning, and meticulous surgical techniques [18].

In conclusion, the dual lobe LDLT is complex and technically challenging procedure and it should be considered a viable option to increase the donor pool and overcome the problem of the small-for-size syndrome.

## Data Availability

Not applicable.
